# Groupwise information sharing promotes ingroup favoritism in indirect reciprocity

**DOI:** 10.1186/1471-2148-12-213

**Published:** 2012-11-05

**Authors:** Mitsuhiro Nakamura, Naoki Masuda

**Affiliations:** 1 Department of Mathematical Informatics, The University of Tokyo, Bunkyo, Tokyo 113-8656, 7-3-1 Hongo, Japan

## Abstract

**Background:**

Indirect reciprocity is a mechanism for cooperation in social dilemma situations. In indirect reciprocity, an individual is motivated to help another to acquire a good reputation and receive help from others afterwards. Another aspect of human cooperation is ingroup favoritism, whereby individuals help members in their own group more often than those in other groups. Ingroup favoritism is a puzzle for the theory of cooperation because it is not easily evolutionarily stable. In the context of indirect reciprocity, ingroup favoritism has been shown to be a consequence of employing a double standard when assigning reputations to ingroup and outgroup members. An example of such a double standard is the situation in which helping an ingroup member is regarded as good, whereas the same action toward an outgroup member is regarded as bad.

**Results:**

We analyze a computational model of indirect reciprocity in which information sharing is conducted groupwise. In our model, individuals play social dilemma games within and across groups, and the information about their reputations is shared within each group. We show that evolutionarily stable ingroup favoritism emerges even if all the players use the same reputation assignment rule regardless of group (i.e., a single standard). Two reputation assignment rules called simple standing and stern judging yield ingroup favoritism; under these rules, cooperation with (defection against) good individuals is regarded as good (bad) and defection against bad individuals is regarded as good. Stern judging induces much stronger ingroup favoritism than does simple standing. Simple standing and stern judging are evolutionarily stable against each other when groups employing different assignment rules compete and the number of groups is sufficiently large. In addition, we analytically show as a limiting case that homogeneous populations of reciprocators that use reputations are unstable when individuals independently infer reputations of individuals, which is consistent with previously reported numerical results.

**Conclusions:**

Our results suggest that ingroup favoritism can be promoted in indirect reciprocity by the groupwise information sharing, in particular under the stern judging assignment rule.

## Background

Behavioral nature of humans depends on the economy of reputations, where praise and blame often lead to gain and loss of material benefits [1,2]. Humans, among other animals, cooperate via indirect reciprocity, which involves cooperation beyond pairwise relationships [3-6]. In indirect reciprocity based on reputations, an individual acquires a good reputation by behaving cooperatively in apposite situations. The cost of maintaining a good reputation is compensated for by other individuals’ future cooperation toward the individual possessing the good reputation. Indirect reciprocity has been extensively studied in both theories [5-19] and experiments [2,20-23].

Another facet of human cooperation is that an individual often cooperates with members in the same group and not with others, a phenomenon called ingroup favoritism [24-33]. Ingroup favoritism poses a puzzle for the theory of cooperation because it is usually not Pareto efficient; i.e., the payoff to an individual in the case of ingroup favoritism is smaller than that in the case of group-independent all-out cooperation. In addition, an individual implementing ingroup favoritism is worse off than an individual defecting against both ingroup and outgroup members unless a specific assumption is imposed. In fact, known mechanisms for stable ingroup favoritism (e.g., correlation between altruistic traits and phenotypic tags [34-36], incomplete observability of tags [37], combination of mutation of tags and limited dispersal [38]) are, in our view, complicated. Otherwise, stable ingroup favoritism requires an additional mechanism (e.g., intergroup conflict [39,40]) that is capable of stabilizing cooperation on its own.

If maintaining a good reputation is a concern, why do individuals want to discriminate between ingroup and outgroup fellows? One of the present authors has shown that ingroup favoritism is evolutionarily stable in various situations when only group-level reputations are available in regard to outgroup members [41]. In the model, an individual’s action changes the individual’s reputation in the eyes of the ingroup members, and the action also changes the reputation of the group to which the individual belongs. It was revealed that the action rule of individuals (i.e., the strategy depending on the reputation of the coplayer) toward ingroup and outgroup members and the reputation assignment rule (also called the social norm) used for evaluating ingroup and outgroup interactions, or at least the latter, must discriminate between ingroup and outgroup members for stabilizing ingroup favoritism. An example is a rule whereby cooperation toward outgroup members is frowned upon, whereas the same behavior toward ingroup members leads to a good reputation. Consistent with this theoretical example, Yamagishi and colleagues had conducted behavioral experiments suggesting that ingroup favoritism occurs because subjects anticipate that the reputation mechanism is functional only inside the group [25-27,29,33]. These theoretical and experimental results suggest that double standards, in terms of the action rule or the reputation assignment rule, may underpin ingroup favoritism.

In the context of indirect reciprocity, group structure may play a crucial role in spreading reputations of individuals via rumor and gossip. In general, individuals interact more frequently with ingroup members than with outgroup members [42]. Therefore, rumor and gossip may enable sharing of reputations of individuals more smoothly within a group than between different groups. Most theoretical studies of indirect reciprocity have assumed that information sharing and interactions occur randomly in a well-mixed population. Otherwise, individuals are assumed to not exchange information about reputations [7,10,13,19].

In the present study, we explore a scenario of ingroup favoritism without resorting to rules that apply double standards. In practice, humans may not differentiate between ingroup and outgroup coplayers with regard to their action rules or reputation assignment rules. We analyze a group-structured model of indirect reciprocity, in which an individual’s reputation is shared by each group but not between groups. We study the case in which all the players use the same reputation assignment rule and the case in which players in different groups use different reputation assignment rules. We show that ingroup favoritism can emerge when players simply implement reputation-based decision making and do not favor ingroup members. Because of the assumed groupwise information sharing and some reputation assignment error, ingroup and outgroup members tend to possess good and bad reputations, respectively, without further assumptions. In particular, ingroup favoritism is strong when individuals adopt a reputation assignment rule called stern judging, under which helping bad individuals is regarded as bad.

## Methods

### Model

We consider an infinitely large population of players divided into *M* (≥ 2) groups. Each group is assumed to contain the equal fraction, 1/*M*, of players. In the population, players are involved in sufficiently many rounds of the so-called donation game. In a one-shot donation game, two players are randomly selected from the population, one as donor and the other as recipient. We assume that the donor and recipient belong to the same group with probability *θ*. The donor cooperates (C), i.e., provides help, or defects (D), i.e., refrains from helping, depending on the donor’s action rule and the recipient’s reputation (good (G) or bad (B)). Action C imposes cost *c* (> 0) on the donor and results in benefit *b* (>* c*) imparted to the recipient. Action D does not change the payoff to either the donor or recipient. A donor adopting action rule ALLC cooperates with any recipient. A donor adopting action rule ALLD defects against any recipient. A donor adopting action rule DISC cooperates with G recipients and defects against B recipients.

**Figure 1:**
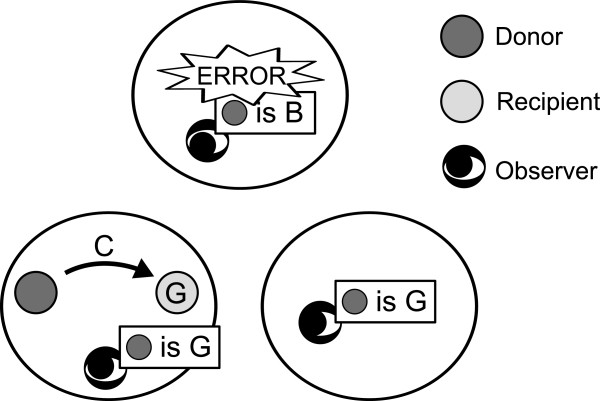
**Behavior of different observers in different groups (*M *= 3).**

To know a recipient’s reputation, the donor consults the unique information source, called the observer, that is shared by the group to which the donor belongs. Therefore, players in different groups may perceive different reputations (i.e., G or B) of the same player. The observer in each group independently assigns a reputation to the donor and shares it with the other players in the observer’s group. Observers intend the predefined reputation assignment toward a donor’s action but may assign a reputation opposite to the intention. The *M* observers independently commit such assignment error with probability *μ*(≪ 1). In the example of intragroup interaction shown in Figure 1, all the three observers intended to assign G to the donor, and one observer erroneously assigned B to the donor. If the assignment error occurs, the “wrong” reputation is shared by all the players in the group to which the observer belongs.

**Figure 2:**
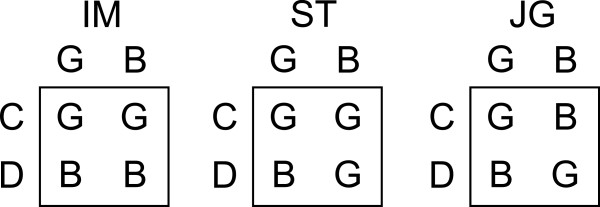
**Three reputation assignment rules.** Image scoring (IM), simple standing (ST), and stern judging (JG). The rows represent the donor’s actions (i.e., C and D), the columns represent the recipient’s reputations (G and B), and G and B inside the boxes represent the reputations that observers assign to the donor.

Observers assign reputations according to a common reputation assignment rule unless otherwise stated. We principally compare three rules: image scoring (IM), simple standing (ST), and stern judging (JG) [5,6,10-12,14,18], symbolically shown in Figure 2. Among the three rules, IM is the simplest rule under which observers assign G and B to a donor that has selected C and D, respectively. ST and JG are simplest among the so-called “leading eight” reputation assignment rules that stabilize cooperation in well-mixed populations [11,12]. Under ST and JG, the new reputation of the donor depends on the action of the donor (i.e., C or D) and the reputation of the recipient (i.e., G or B). When a recipient has a G reputation, observers assign G and B to a C and D donor, respectively, under both ST and JG. When a recipient has a B reputation, observers assign G to a D donor under both rules. The two rules are different in that helping bad individuals (i.e., a donor’s C with a B recipient) is appreciated (i.e., G imparted by the observer) under ST, whereas the same action of the donor is punished (i.e., B imparted by the observer) under JG; JG is sterner than ST [1,18].

After sufficiently many rounds of the donation game involving reputation updates, the reputation distribution in the eyes of each group-specific observer reaches a unique equilibrium. In the equilibrium, we measure the quantities of interest such as the fractions of G players, the probability of cooperation, and their dependence on groups.

### Analysis methods

#### Equilibria of the reputation dynamics

Table 1 summarizes the definitions of the symbols used in this section.

**Table 1:** Meaning of symbols

**Symbol**	**Meaning**
*M*	Number of groups
*θ*	Probability that a donor and recipient in a one-shot game are in the same group
***r***∈{G, B}^*M*^	Reputation vector of a player in the eyes of *M* observers
*p*_*k*_(***r***)	Probability that a player in group *k* has reputation vector ***r***
*p*_−*k*_(***r***)	Probability that a player outside group *k* has reputation vector ***r***
*σ*(*r*)∈{C, D}	Donor’s action to a recipient having reputation *r*∈{G, B}
Φ_*r*_(*a*,*r*^*′*^)	Probability that an observer assigns reputation *r*∈{G, B} to a donor selecting action *a*∈{C, D} to a recipient having reputation *r*^*′*^∈{C, D}
*p*_in_(*r*)	Probability that a player in the eyes of an ingroup observer has reputation *r*
*p*_out_(*r*)	Probability that a player in the eyes of an outgroup observer has reputation *r*

We examine the stability of a homogeneous population of DISC players. Each player bears a reputation vector, ***r***=(*r*_1_*r*_2_,…,*r*_*M*_)∈{G,*B*}^*M*^, in the eyes of *M* observers, each representing a group. We denote by *p*_*k*_(***r***) the probability that a player in group *k* has reputation vector ***r***. By adopting the formalism developed by Ohtsuki & Iwasa [11], we obtain the following reputation dynamics:

(1) $$\begin{array}{l} \frac{d}{dt}p_{k}(r)=-\;p_{k}(r)+\underset{r^{\prime }\in {\left\{G,B\right\}}^{M}}{\sum }\left\{\theta p_{k}(r^{\prime })+(1-\theta )p_{-k}(r^{\prime })\right\}\\ \;\;\times \overset{M}{\underset{k^{\prime }=1}{\prod }}\Phi_{r_{k^{\prime }}}(\sigma (r_{k}^{\prime }),r_{k^{\prime }}^{\prime }). \end{array}$$

The summation on the right-hand side of Eq. (1) represents the average over the recipient’s reputation vector ***r***^*′*^. With probability *θ*, a game involves a donor and a recipient in group *k*, and the recipient has reputation vector ***r***^*′*^ with probability *p*_*k*_(***r***^*′*^). With probability 1−*θ*, a donor and a recipient belong to group *k* and another group, respectively, and the recipient has reputation vector ***r***^*′*^ with probability $p_{-k}(r^{\prime })\equiv \overset{M}{\underset{k^{\prime }=1,k^{\prime }\neq k}{\sum }}p_{k^{\prime }}(r^{\prime })/(M-1)$. $\sigma (r_{k}^{\prime })$ represents a donor’s action toward a recipient having reputation $r_{k}^{\prime }$. Because we assume DISC donors, *σ*(G) = C and *σ*(B) = D. The reputation assignment rule is essentially given by $\Phi_{r_{k^{\prime }}}(\sigma (r_{k}^{\prime }),r_{k^{\prime }}^{\prime })\in \{1-\mu ,\mu \}$, which is the probability that an observer in group *k*^*′*^assigns reputation $r_{k^{\prime }}$ to a donor in group *k*. This probability depends on the donor’s action $\sigma (r_{k}^{\prime })$ toward a recipient having reputation $r_{k^{\prime }}^{\prime }$ in the eyes of the observer in each group *k*^*′*^. In Table 2, we list the Φ values under different assignment rules. It should be noted that all the observers use a unique assignment rule unless otherwise stated; we do not basically assume that observers employ different assignment rules as in previous studies [10,15,16,18,19].

**Table 2:** Probability that an observer assigns G to a donor

**Rule**	**Φ**_**G**_**(C, G)**	**Φ**_**G**_**(D, G)**	**Φ**_**G**_**(C, B)**	**Φ**_**G**_**(D, B)**
IM	1−*μ*	*μ*	1−*μ*	*μ*
ST	1−*μ*	*μ*	1−*μ*	1−*μ*
JG	1−*μ*	*μ*	*μ*	1−*μ*

Φ_G_(*a*,*r*) represents the probability that a donor receives G when the donor selects action *a*∈{C, D} and the recipient has reputation *r*∈{G, B}. The donor receives B with probability Φ_B_(*a*,*r*) = 1−Φ_G_(*a*,*r*).

**Figure 3:**
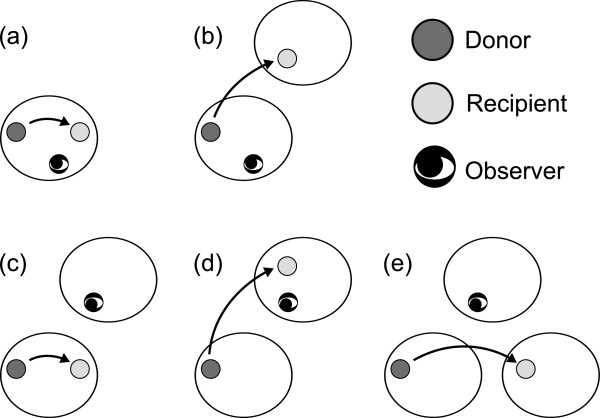
**Five possible situations of the reputation update.** Observations are made by ingroup observers in **(a)** and **(b)**, and by outgroup observers in **(c)**, **(d)**, and **(e)**.

We reduce Eq. (1) to mean field dynamics of two reputation distributions. First, we apply summation $\underset{r_{-k}}{\sum }\equiv \underset{r_{1}}{\sum }\underset{r_{2}}{\sum }\ldots \underset{r_{k-1}}{\sum }\underset{r_{k+1}}{\sum }\ldots \underset{r_{M}}{\sum }$ to both sides of Eq. (1) to obtain the reputation dynamics in the eyes of ingroup observers as follows:

(2) $$\;\begin{array}{l} \frac{d}{dt}p_{\mathrm{in}}(r)=-\;p_{\mathrm{in}}(r)+\underset{r^{\prime }\in \{G,B\}}{\sum }\left\{\theta p_{\mathrm{in}}(r^{\prime })+(1-\theta )p_{\text{out}}(r^{\prime })\right\}\\ \;\;\times \Phi_{r}(\sigma (r^{\prime }),r^{\prime }), \end{array}$$

where $p_{\mathrm{in}}(r)\equiv \underset{r_{-k}}{\sum }p_{k}(r)$ and $p_{\text{out}}(r)\equiv \underset{r_{-k}}{\sum }p_{-k}(r)$ are the probabilities that a player has reputation *r*∈{G,*B*} in the eyes of ingroup and outgroup observers, respectively. The two terms inside the curly brackets on the right-hand side of Eq. (2) correspond to the two situations shown in Figure 3(a) and **(b)**. With probability *θp*_in_(*r*^*′*^), the recipient belongs to the donor and observer’s group, and has reputation *r*^*′*^ (Figure 3(a)). With probability (1−*θ*)*p*_out_(*r*^*′*^), the recipient does not belong to the donor and observer’s group, and has reputation *r*^*′*^(Figure 3(b)).

Second, by applying summation $\underset{r_{-\ell }}{\sum }\equiv \underset{r_{1}}{\sum }\underset{r_{2}}{\sum }\ldots$$\underset{r_{\ell -1}}{\sum }\underset{r_{\ell +1}}{\sum }\ldots \underset{r_{M}}{\sum }$, where *ℓ *≠* k*, to both sides of Eq. (1), we obtain

(3) $$\begin{array}{l} \frac{d}{dt}p_{k}(r_{\ell })=-\;p_{k}(r_{\ell })\;+\;\underset{r_{k}^{\prime }\in \{G,B\}}{\sum }\underset{r_{\ell }^{\prime }\in \{G,B\}}{\sum }\left\{\;\theta p_{k}(r_{k}^{\prime },r_{\ell }^{\prime })\;+\;(1\;-\;\theta )\right)\\ \;\;\times \left[\frac{1}{M-1}\;p_{\ell }(r_{k}^{\prime },r_{\ell }^{\prime })+\left(1-\frac{1}{M-1}\right)\right)\\ \;\;\times \left(p_{-k\ell }(r_{k}^{\prime },r_{\ell }^{\prime })]\right\}\Phi_{r_{\ell }}(\sigma (r_{k}^{\prime }),r_{\ell }^{\prime }), \end{array}$$

where $p_{k}(r_{k}^{\prime },r_{\ell }^{\prime })$, $p_{\ell }(r_{k}^{\prime },r_{\ell }^{\prime })$, and $p_{-k\ell }(r_{k}^{\prime },r_{\ell }^{\prime })$ are the probabilities that a player in group *k*, group *ℓ*, and a group other than *k* and *ℓ*, respectively, has reputation $r_{k}^{\prime }$ and $r_{\ell }^{\prime }$ in the eyes of observers in groups *k* and *ℓ*. By approximating the three probabilities by $p_{\mathrm{in}}(r_{k}^{\prime })p_{\text{out}}(r_{\ell }^{\prime })$, $p_{\text{out}}(r_{k}^{\prime })p_{\mathrm{in}}(r_{\ell }^{\prime })$, and $p_{\text{out}}(r_{k}^{\prime })p_{\text{out}}(r_{\ell }^{\prime })$, respectively, we obtain the mean-field reputation dynamics in the eyes of outgroup observers as follows:

(4) $$\begin{array}{l} \frac{d}{dt}p_{\text{out}}(r)=-\;p_{\text{out}}(r)\;+\underset{r^{\prime }\in \{G,B\}}{\sum }\underset{r^{\prime \prime }\in \{G,B\}}{\sum }\left\{\theta p_{\mathrm{in}}(r^{\prime })p_{\text{out}}(r^{\prime \prime })\right)\\ \;\;\;+(1-\theta )\left[\frac{1}{M-1}\;p_{\text{out}}(r^{\prime })p_{\mathrm{in}}(r^{\prime \prime })\right)\\ \;\;\;+\left(\left(\left(1-\frac{1}{M-1}\right)\;p_{\text{out}}(r^{\prime })p_{\text{out}}(r^{\prime \prime })\right]\right\}\\ \;\;\;\times \Phi_{r}(\sigma (r^{\prime }),r^{\prime \prime }). \end{array}$$

The three terms inside the curly brackets on the right-hand side of Eq. (4) correspond to the three situations shown in Figure 3(c), **(d)**, and **(e)**. With probability *θp*_in_(*r*^*′*^)*p*_out_(*r*^*′′*^), the recipient belongs to the donor’s group, which differs from the observer’s group, and has reputation *r*^*′*^ and *r*^*′′*^in the eyes of the donor and observer, respectively (Figure 3(c)). With probability (1−*θ*)[1/(*M*−1)]*p*_out_(*r*^*′*^)*p*_in_(*r*^*′′*^), the recipient belongs to the observer’s group, which differs from the donor’s group, and has reputation *r*^*′*^ and *r*^*′′*^in the eyes of the donor and observer, respectively (Figure 3(d)). With probability (1−*θ*)[1−1/(*M*−1)]*p*_out_(*r*^*′*^)*p*_out_(*r*^*′′*^), the recipient belongs to a group different from the donor’s and observer’s groups, and has reputation *r*^*′*^ and *r*^*′′*^ in the eyes of the donor and observer, respectively (Figure 3(e)).

By setting d*p*_in_(*r*)/d*t *=d* p*_out_(*r*)/d*t *= 0 in Eqs. (2) and (4), we identify stationary points that are candidates of stable equilibria of the reputation dynamics. We examine the conditions det***J ***> 0 and Tr***J ***< 0, where ***J*** is the Jacobian matrix, at each stationary point to identify all the stable equilibria. We confirmed that the stable equilibrium denoted by $p_{\mathrm{in}}^{*}(r)$ and $p_{\text{out}}^{*}(r)$ is unique under each reputation assignment rule.

#### Stability against invasion by ALLC and ALLD mutants

We check the evolutionary stability of a homogeneous population composed of DISC players against invasion by an infinitesimal fraction of mutants adopting ALLC or ALLD. The payoff to a DISC resident player is given by

(5) $$\Pi_{\text{DISC}}=(b-c)\left[\theta p_{\mathrm{in}}^{*}(G)+(1-\theta )p_{\text{out}}^{*}(G)\right],$$

and those to ALLC and ALLD mutants are given by

(6) $$\Pi_{\text{ALLC}}=-c+b\left[\theta p_{\mathrm{in}}^{C}(G)+(1-\theta )p_{\text{out}}^{C}(G)\right]$$

and

(7) $$\Pi_{\text{ALLD}}=b\left[\theta p_{\mathrm{in}}^{D}(G)+(1-\theta )p_{\text{out}}^{D}(G)\right],$$

respectively. In Eqs. (6) and (7), $p_{\mathrm{in}}^{C}(G)$, $p_{\text{out}}^{C}(G)$, $p_{\mathrm{in}}^{D}(G)$, and $p_{\text{out}}^{D}(G)$ represent the probabilities that the mutants selecting C and D acquire G reputations in the eyes of ingroup and outgroup observers, and are given by

$$p_{\mathrm{in}}^{a}(G)=\underset{r^{\prime }\in \{G,B\}}{\sum }\left\{\theta p_{\mathrm{in}}^{*}(r^{\prime })+(1-\theta )p_{\text{out}}^{*}(r^{\prime })\right\}\Phi_{G}(a,r^{\prime })$$

and

$$\begin{array}{l} p_{\text{out}}^{a}(G)=\underset{r^{\prime }\in \{G,B\}}{\sum }\underset{r^{\prime \prime }\in \{G,B\}}{\sum }\left\{\;\theta p_{\mathrm{in}}^{*}(r^{\prime })p_{\text{out}}^{*}(r^{\prime \prime })+(1-\theta )\right)\\ \;\;\times \left[\frac{1}{M-1}p_{\text{out}}^{*}(r^{\prime })p_{\mathrm{in}}^{*}(r^{\prime \prime })+\left(1-\frac{1}{M-1}\right)\right)\\ \;\;\times \left(\left(p_{\text{out}}^{*}(r^{\prime })p_{\text{out}}^{*}(r^{\prime \prime })\;\right]\right\}\Phi_{G}(a,r^{\prime \prime }), \end{array}$$

where *a *= C or D. The population of DISC players is stable against invasion by ALLC and ALLD mutants if

(8) $$\Pi_{\text{DISC}}>\max\left\{\Pi_{\text{ALLC}},\Pi_{\text{ALLD}}\right\}.$$

#### Cooperativeness

DISC donors cooperate exclusively with G recipients. Therefore, in each stable equilibrium, the probability of cooperation, which we call the cooperativeness, toward ingroup and outgroup recipients is given by $p_{\mathrm{in}}^{*}(G)$ and $p_{\text{out}}^{*}(G)$, respectively. The cooperativeness for the entire population is given by

(9) $$\psi \equiv \theta p_{\mathrm{in}}^{*}(G)+(1-\theta )p_{\text{out}}^{*}(G).$$

#### Measurement of ingroup bias

To quantify the degree of ingroup bias, we measure the difference between ingroup and outgroup cooperativeness, defined by

(10) $$\rho \equiv p_{\mathrm{in}}^{*}(G)-p_{\text{out}}^{*}(G).$$

When *ρ *≈ −1, players basically cooperate with outgroup recipients and defect against ingroup recipients, implying outgroup favoritism. When *ρ *≈ 0, players equally likely cooperate with ingroup and outgroup recipients. When *ρ *≈ 1, players cooperate with ingroup recipients and defect against outgroup recipients, implying ingroup favoritism.

## Results

Table 3 summarizes the results obtained under the three reputation assignment rules.It shows the stable fractions of G players in the eyes of ingroup and outgroup observers (i.e., $p_{\mathrm{in}}^{*}(G)$ and $p_{\text{out}}^{*}(G)$), the stability conditions, cooperativeness (i.e., *ψ*), and the degree of ingroup bias (i.e., *ρ*).

**Table 3:** Equilibria and the stability conditions for a population of DISC players under different assignment rules

**Rule**	$p_{\text{in}}^{*}(\text{G})$	$p_{\text{out}}^{*}(\text{G})$	**Stability condition**	***ψ***	***ρ***
IM	$\frac{1}{2}$	$\frac{1}{2}$	Unstable	$\frac{1}{2}$	0
ST	1−*μ*	$1-\mu \frac{1+\theta }{\theta }+O(\mu^{2})$	Eq. (14)	$1-\frac{\mu }{\theta }+O(\mu^{2})$	$\frac{\mu }{\theta }+O(\mu^{2})$
JG	1−*μ*	$\frac{1}{2}$	Eq. (18)	$\frac{1+\theta }{2}-\mu \theta$	$\frac{1}{2}-\mu$

### IM

Under IM, the equilibrium fractions of G players in the eyes of ingroup and outgroup observers are both equal to *ψ *= 1/2. Therefore, ingroup favoritism does not occur, i.e., *ρ *= 0. Furthermore, the population of DISC players is invaded by ALLC mutants such that it is unstable. This result is consistent with the established result that cooperation is usually unstable under IM because observers do not distinguish between selfish defection (i.e., D against G recipients) and justified defection (i.e., D against B recipients) [1,8,10,43].

### ST

Under ST, DISC players almost always cooperate with ingroup recipients, i.e., $p_{\mathrm{in}}^{*}(G)=1-\mu$. This result is consistent with the previous results in which ST enables perfect cooperation when a population does not possess group structure (corresponding to *M *= 1) [11,12].

The fraction of G players in the eyes of outgroup observers is given by

(11) $$p_{\text{out}}^{*}(G)=1-\mu \frac{1+\theta }{\theta }+O(\mu^{2}).$$

Therefore, DISC players almost always cooperate with both ingroup and outgroup recipients unless *θ* is small (i.e., *ψ *= 1−*μ*/*θ* + *O*(*μ*^2^)). Because donors defect slightly more often against outgroup than ingroup recipients, weak ingroup favoritism occurs (i.e., *ρ *=* μ*/*θ* + *O*(*μ*^2^)).

Equations (5), (6), and (7) yield the payoff differences given by

(12) $$\Pi_{\text{ALLC}}-\Pi_{\text{DISC}}=\frac{\mu }{\theta }\left[b(1-\theta )-c\right]+O(\mu^{2})$$

and

(13) $$\Pi_{\text{ALLD}}-\Pi_{\text{DISC}}=-(b-c)+O(\mu ).$$

Therefore, the stability condition (Eq. (8)) reads

(14) $$1<\frac{b}{c}<\frac{1}{1-\theta }.$$

ALLC mutants invade DISC players if *b*/*c *> 1/(1−*θ*). The cooperation is stable up to a large value of *b*/*c* when ingroup interaction is frequent (i.e., large *θ*). ALLD mutants invade a DISC population under a trivial condition *b*/*c *< 1.

### JG

Under JG, DISC players have the same cooperativeness toward ingroup recipients as under ST, i.e., $p_{\mathrm{in}}^{*}(G)=1-\mu$. This result is consistent with the previous results in which JG enables perfect cooperation when a population does not possess group structure (corresponding to *M *= 1) [11,12].

The fraction of G players in the eyes of outgroup observers is given by

(15) $$p_{\text{out}}^{*}(G)=\frac{1}{2}.$$

Therefore, DISC players cooperate with outgroup recipients with probability 1/2. In contrast to the case of ST, frequent intergroup interaction considerably reduces cooperation under JG (i.e., *ψ *= (1 + *θ*)/2 + *μθ*). The degree of ingroup bias under JG is given by *ρ *= 1/2−*μ*, which is independent of *θ*. DISC players show a significant level of ingroup favoritism, even though they simply use the reputations without intending to discriminate recipients by the group identity.

The payoff differences are given by

(16) $$\;\Pi_{\text{ALLC}}-\Pi_{\text{DISC}}=-\frac{1-\theta }{2}\left[b\frac{M\theta -1}{M-1}+c\right]+O(\mu )$$

and

(17) $$\begin{array}{l} \Pi_{\text{ALLD}}\;-\;\Pi_{\text{DISC}}=-\frac{1}{2}\left[b\frac{1+(M-3)\theta \;+\;M\theta^{2}}{M-1}\;-\;c(1+\theta )\right]\\ \;\;\;\;+O(\mu ). \end{array}$$

The stability condition reads

(18) $$\left\{\begin{array}{ll} \frac{\left(M-1)(1+\theta \right)}{1+(M-3)\theta +M\theta^{2}}<\frac{b}{c}<\frac{M-1}{1-M\theta } & \text{if}\;\;0\leq \theta <\frac{1}{M},\\ \frac{\left(M-1)(1+\theta \right)}{1+(M-3)\theta +M\theta^{2}}<\frac{b}{c} & \text{if}\;\;\frac{1}{M}\leq \theta \leq 1. \end{array}\right)$$

The DISC population is resistant to invasion by ALLC mutants when *θ *≥ 1/*M*, i.e., when ingroup interaction occurs more frequently than in the case of unbiased random pairing. When *θ *< 1/*M* and *b*/*c *> (*M*−1)/(1−*Mθ*), ALLC mutants invade the population of DISC players. When *b*/*c *< (*M*−1)(1 + *θ*)/[1 + (*M*−3)*θ* + *Mθ*^2^, ALLD mutants invade the population of DISC players. The cooperation is stable down to a small value of *b*/*c* if ingroup interaction is frequent (i.e., large *θ*) or the number of groups (i.e., *M*) is small. In the limit *M* → ∞, Eq. (18) is reduced to *b*/*c *> 1/*θ*, which coincides with the results obtained from a previous model with infinite groups [41].

Under both ST and JG, in particular JG, ingroup favoritism emerges. This is because the donors (equivalently, ingroup observers) and outgroup observers generally perceive different reputations of the same player due to the assignment error (see Figures 1, 3(c), 3**(d)**, and 3**(e)**). For example, if a donor defects against a recipient whose reputation is B in the eyes of the donor’s group members, the donor receives a G reputation from the ingroup observer. However, if the same recipient has a G reputation in the eyes of the outgroup observer, the outgroup observer assigns B to the donor under ST and JG. As another example, if a donor cooperates with a recipient whose reputation is G in the eyes of the donor’s group members, the donor receives G from the ingroup observer. However, if the recipient has a B reputation in the eyes of the outgroup observer, the outgroup observer assigns B to the donor under JG. As these examples suggest, different groups may perceive the opposite reputations of the same players in a long run. Players in the same group coordinate the subjective information about a given player’s reputation, whereas those in different groups do not. This discrepancy causes ingroup favoritism.

### Numerical results

**Figure 4:**
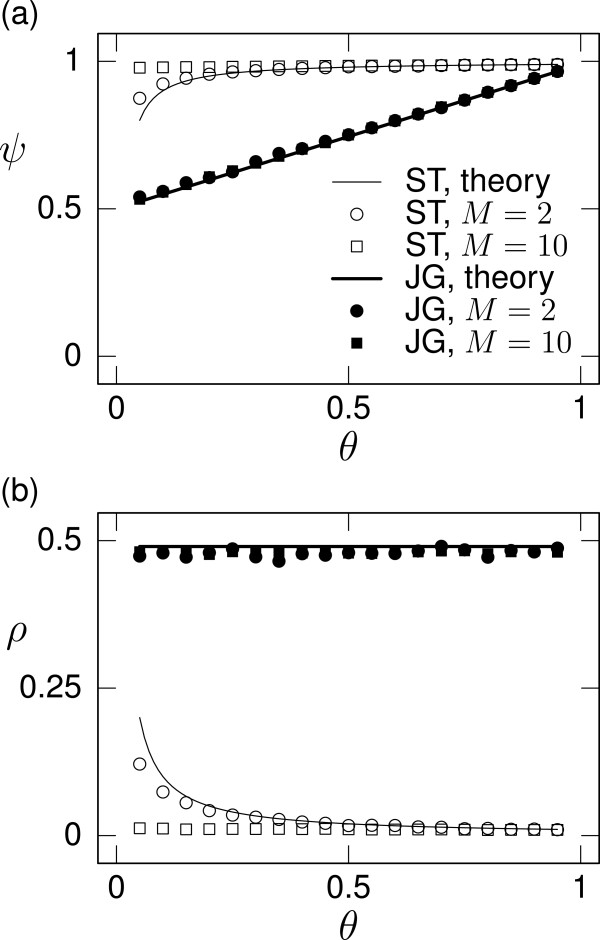
**Equilibria for a population of DISC players under ST and JG. (a)** Cooperativeness (*ψ*) and **(b)** ingroup bias (*ρ*). We vary the assignment rule (ST or JG), the number of groups (*M *= 2 or 10), and the probability of ingroup interaction (*θ*). The lines represent theoretical results shown in Table 3. The symbols represent numerical results.

We compare the theoretical results with numerical results obtained from individual-based simulations in Figure 4. The procedure of the numerical analysis is described in Appendix A Numerical methods in the case of the homogeneous assignment rule. The analytical and numerical results are sufficiently close to each other in terms of both cooperativeness (Figure 4(a)) and ingroup bias (Figure 4(b)).

**Figure 5:**
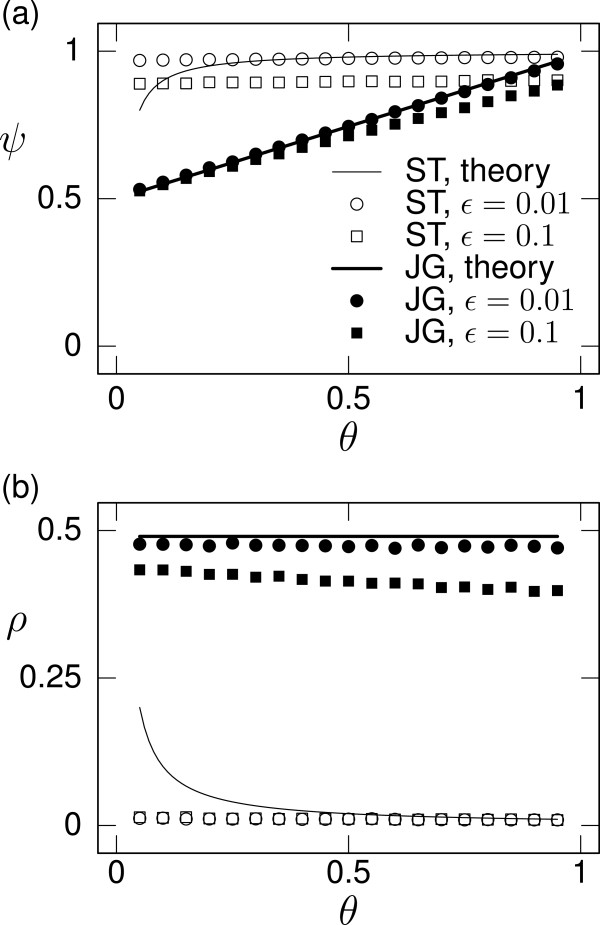
**Equilibria for a population of DISC players under action implementation error. (a)** Cooperativeness (*ψ*) and **(b)** ingroup bias (*ρ*). We fix the number of groups (*M *= 10) and vary the assignment rule (ST or JG), the probability that a donor fails to help a recipient (*ε *= 0.01 or 0.1), and the probability of ingroup interaction (*θ*). The lines represent theoretical results when *ε *= 0 and are the replicates of those shown in Table 3. The symbols represent numerical results.

We also examine the error-prone case in which donors fail to help recipients (i.e., select D when the donors intend C) with probability *ε*[9]. The numerical results for *ε *= 0.01 and 0.1 are shown in Figure 5. The error reduces cooperativeness (Figure 5(a)) and ingroup bias (Figure 5(b)) under both ST and JG (see Figure 4 for the error-free case). Nevertheless, the results with the error are qualitatively the same as those without the error.

### Mixed assignment rules

We have shown that JG leads to strong ingroup favoritism, whereas ST does not. To examine the transition between the two regimes, we consider an assignment rule denoted by MX, which is a mixture of JG and ST. In a one-shot game under MX, observers independently assign reputations by using JG with probability *α* and ST with probability 1−*α*. Therefore, Φ_G_(C,*G*) = 1−*μ*, Φ_G_(D,*G*) =* μ*, Φ_G_(C,*B*) =* αμ* + (1−*α*)(1−*μ*), and Φ_G_(D,*B*) = 1−*μ*. Parameter *α* controls the degree of sternness with which observers assign B to donors that cooperate with B recipients. ST and JG correspond to *α *= 0 and *α*=1, respectively. We numerically solve Eqs. (2) and (4) under MX.

**Figure 6:**
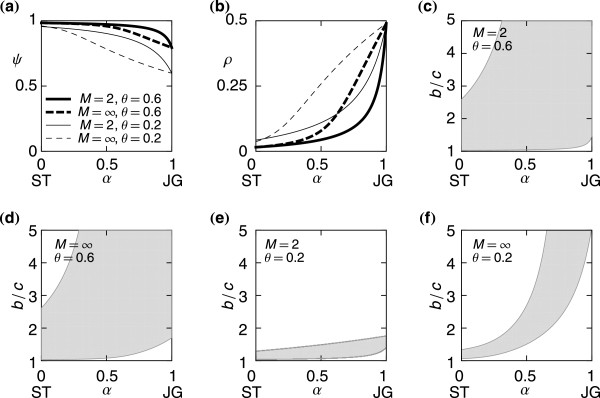
**Equilibria and the stability conditions for a population of DISC players under MX. (a)** Cooperativeness (*ψ*) and **(b)** ingroup bias (*ρ*). In **(a)** and **(b)**, we set (*M*,*θ*) = (2,0.6),(∞,0.6), (2,0.2), and(∞,0.2). **(c)**–**(f)** Stability conditions. The homogeneous population of DISC players is stable in the shaded parameter regions. We set **(c)**(*M*,*θ*) = (2,0.6), **(d)**(*M*,*θ*) = (∞,0.6), **(e)**(*M*,*θ*)=(2,0.2), and **(f)**(*M*,*θ*) = (∞,0.2).

The results under MX are shown in Figure 6. Sternness gradually decreases cooperativeness (Figure 6(a)) and increases ingroup bias (Figure 6(b)) for different values of *M* and *θ*. The results interpolate those for ST and JG and imply that sternness promotes ingroup favoritism. The shaded parameter regions in Figure 6(c)–**(f)** indicate the values of *α *and *b*/*c *for which DISC residents are stable. Above (below) the shaded regions, ALLC (ALLD) mutants invade the DISC population. In all the cases, the upper and lower bounds of the stability region in terms of *b*/*c* increase with *α*. A decrease in *M* induces cooperativeness and reduces ingroup bias. A decrease in *M* also broadens the stability regions if *θ* is large. An increase in *θ *induces cooperativeness, reduces ingroup bias, and broadens the stability regions for the following reason. When *θ* is large, players are largely involved in ingroup interactions. Then, they do not suffer from a B reputation that outgroup observers may frequently attach to the donor because of the discrepancy between players’ reputations perceived by different groups (see subsection JG in Results for related discussion).

### Heterogeneous assignment rules

We have assumed that all the groups use a common reputation assignment rule. In this section, we numerically examine a case in which observers in different groups use different reputation assignment rules. We consider a situation in which *m* (1 ≤* m *≤* M *− 1) groups use JG and *M*−*m *groups use ST. The procedure of the numerical analysis is described in Appendix B Numerical methods in the case of the heterogeneous assignment rule.

**Figure 7:**
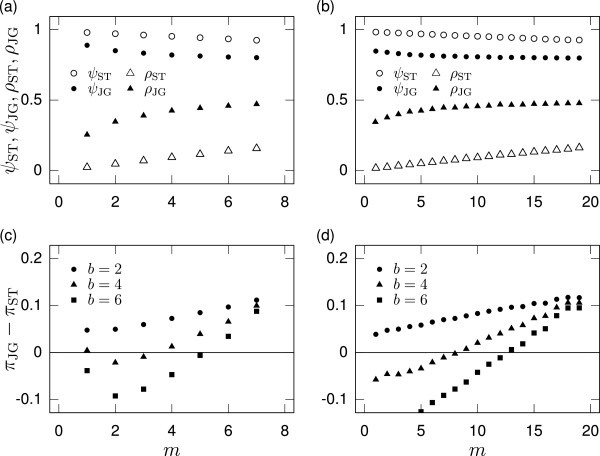
**Equilibria for a population of DISC players under heterogeneous assignment rules. (a)**, **(b)** Cooperativeness (*ψ*_ST_and *ψ*_JG_) and ingroup bias (*ρ*_ST_ and *ρ*_JG_) for groups employing ST and JG. **(c)**, **(d)** Payoff difference between a player in a ST group and that in a JG group (*Π*_JG_−*Π*_ST_). We set *θ *= 0.6 and *c *= 1. We also set *M *= 8 in **(a)** and **(c)**, *M *= 20 in **(b)** and **(d)**, and vary the number of JG groups (i.e., *m*) and *b*.

Numerically obtained equilibria with *M *= 8 and *M *= 20 are shown in Figure 7(a) and **(b)**, respectively. As the number of JG groups (i.e., *m*) increases, the cooperativeness (*ψ*_ST_ and *ψ*_JG_ for ST and JG groups, respectively) decreases, and ingroup bias (*ρ*_ST_ and *ρ*_JG_ for ST and JG groups, respectively) increases. Figure 7(c) and **(d)** shows the difference between the payoff to a player in a ST group and that to a player in a JG group (i.e., *Π*_JG_−*Π*_ST_) when *M *= 8 and *M *= 20, respectively. When the benefit-to-cost ratio is small (i.e., *b *= 2), *Π*_JG_−*Π*_ST_ is positive. Therefore, if observers update their assignment rules according to an evolutionary dynamics (e.g., group competition [16]), the evolutionary dynamics would lead to a homogeneous population in which all the observers adopt JG. When the benefit-to-cost ratio is large (i.e., *b *= 6), *Π*_JG_−*Π*_ST_ is positive when *m* is large and negative when *m* is small. This implies that a homogeneous population of ST and that of JG are bistable under evolutionary dynamics. The basin of attraction for the homogeneous ST population in terms of *m* broadens as *b* increases. When the benefit-to-cost ratio takes an intermediate value (i.e., *b *= 4), the results for *M *= 8 (Figure 7(c)) and those for *M *= 20 (Figure 7(d)) are qualitatively different. For *M *= 8, *Π*_JG_−*Π*_ST_ is negative only when *m *= 2 or 3. Therefore, a stable mixture of ST and JG groups and a homogeneous population of JG are bistable. For *M *= 20, a homogeneous population of ST and that of JG are bistable.

## Discussion

In the present study, we showed that ingroup favoritism emerges in a group-structured model of indirect reciprocity. In our model, players share information about reputations in each group but not across different groups. We assumed that a player’s action purely depends on the coplayer’s reputation; players do not refer to the group identity of the coplayers or use other types of prejudice. We also assumed that observers impartially assess ingroup and outgroup donors. We analyzed the model using a mean-field approximation and numerical simulations. Ingroup favoritism occurs under both simple standing (ST) and stern judging (JG) assignment rules. The cooperativeness is reduced by the frequent intergroup interactions, i.e., small *θ*. The ingroup bias is severer and the cooperativeness is smaller under JG than under ST. The parameter region for the stability of the cooperative equilibrium is larger under JG than under ST. Under ST and JG, a population of discriminators is evolutionarily stable if the probability of ingroup interaction (i.e., *θ*) is sufficiently large. If *θ*is small, the population is invaded by unconditional cooperators and unconditional defectors under ST and JG, respectively. We also studied the case in which observers may adopt different assignment rules in different groups. We found that JG would dominate ST in evolutionary settings when the benefit-to-cost ratio is small. Otherwise, the homogeneous population in which all the groups employ ST and that in which all the groups employ JG are bistable in large parameter regions.

Different mechanisms govern ingroup favoritism in our model and that observed in psychological experiments [24-33]. In the latter, players use a cue that indicates the group identity of the coplayer and preferably cooperate with ingroup members. In our model, players do not refer to the group identity of the coplayer. They show ingroup favoritism because they perceive that outgroup members have bad reputations more often than do ingroup members.

We implemented the group structure by controlling probabilities of ingroup and outgroup interactions (i.e., *θ* and 1−*θ*, respectively) and assuming the groupwise information sharing. In terms of the structure of information sharing, most previous theoretical studies of indirect reciprocity are classified into two types: public [5,6,8,9,11,12,14-17,41] and private [7,10,13,17,19] reputation models.

In public reputation models, all the players have access to a common information source that provides the reputation values of the players. Therefore, a donor and observer perceive the same reputation of a recipient such that they do not suffer from the discrepancy of reputations. In public reputation models without group structure of the population, ST and JG realize evolutionarily stable cooperation [11,12]. This result is consistent with ours because, in the limit *θ*→1, Eqs. (14) and (18) are reduced to a trivial condition *b*/*c *> 1 such that the population of discriminators is stable under ST and JG.

In private reputation models, each player individually collects others’ reputations such that a reputation of a player varies between individuals. In contrast to the case of public reputation models, a homogeneous population of discriminators is invaded by unconditional cooperators in private reputation models. A mixture of discriminators and unconditional cooperators is often stable under variants of ST [7,10,13,19]. Under variants of JG, a population of discriminators is invaded by unconditional defectors [17,19] (but see Ref. [13]), or discriminators and unconditional cooperators are frequent in an island model if dispersal of offspring is confined within each island [10]. In the limit *θ*→0 and *M* → ∞, our model can be interpreted as a private reputation model. In this situation, the population of discriminators is unstable because Eqs. (14) and (18) are violated. Therefore, the results obtained from our model in this limit are consistent with the previous results.

For intermediate *θ *and *M* values, our model uses a public reputation scheme within each group and a private reputation scheme across groups. In this sense, the structure of information sharing in our model is situated between public and private reputation models.

One of the present authors previously studied a model of ingroup favoritism on the basis of indirect reciprocity [41], which we refer to as the multiple standard model. The multiple standard model and the model analyzed in the present study are different in two aspects. First, in the multiple standard model, a given player’s reputation is made public to different groups such that the problem of coordination in regard to reputations among different groups does not exist. In the present model, observers in different groups may differently perceive a player’s reputation, which leads to the coordination problem. Second, in the multiple standard model, observers are allowed to use different rules to assign reputations to ingroup and outgroup members. Similarly, donors may use different action selection rules toward ingroup and outgroup recipients. Then, ingroup favoritism of different degrees emerges. Consider a situation in which the action rule is of a single standard such that donors are discriminators toward both ingroup and outgroup recipients. Then, at most partial ingroup favoritism in which players always cooperate with ingroup members and partially (i.e., with probability 1/2) cooperate with outgroup members is evolutionarily stable. Consider another situation in which the action rule is of a double standard such that donors are discriminators toward ingroup members and unconditional defectors toward outgroup members. Then, perfect ingroup favoritism in which players always cooperate with ingroup members and always defect against outgroup members is evolutionarily stable. In the present model, observers use a single-standard reputation assignment rule, and donors use a single-standard action rule. Then, partial ingroup favoritism, but not perfect ingroup favoritism, can be evolutionarily stable.

Group competition models of indirect reciprocity were previously studied [15,16]. In references [15,16], the authors numerically examined competition between different assignment rules employed in different groups. In our terminology, they assumed that the donation game is played inside each group and that reputations are updated exclusively by ingroup observers under the public reputation scheme. They showed that JG (stern-judging in their terminology) emerges in the course of evolutionary dynamics based on group competition and individual selection. Their models and ours are fundamentally different although both studies have stressed the importance of JG. First, they assumed group competition and we did not. Second, they mainly focused on competition between different assignment rules and we did not; we only studied the special case in which observers in different groups adopt either of ST or JG. Third, we determined the possibility of ingroup favoritism and group-independent cooperation. In contrast, their model is not concerned with ingroup favoritism because interaction between a donor and recipient in different groups is not assumed.

Uchida and Sigmund analyzed competition between assignment rules by using replicator dynamics [18]. In their model, a player selected as donor uses the public information source corresponding to the assignment rule that the player adopts. For example, if the surviving assignment rules are only ST and JG (SUGDEN and KANDORI, respectively, in their terminology), there are two public information sources. Although their model is apparently a public reputation model, the players can be interpreted to belong to one of the groups defined by the assignment rule; members in each group share a common information source and use the same assignment rule. Helping a recipient having a bad reputation in the eyes of both ST and JG groups is assessed to be good by the ST group and bad by the JG group. Therefore, JG players assess ST players to be bad more often than they assess JG players. Because this tendency is strong enough, ingroup favoritism occurs in the JG group. Their model and ours are consistent with each other because, when different groups can adopt different assignment rules, both their model and ours with sufficiently many groups predict bistability between ST and JG. Their model and ours complement each other in the following respects. First, they investigated competition between assignment rules, whereas we mainly studied the case in which all the groups share an assignment rule. Second, they assumed a well-mixed population, whereas we varied the frequency of ingroup and outgroup interactions. Third, they studied competition among at most five groups (i.e., five assignment rules), whereas we assumed a general number of groups.

## Conclusion

To explore the possibility of spontaneous ingroup favoritism in indirect reciprocity, we analyzed a social dilemma game in a population with group structure. We showed that the degree of ingroup bias depends on the reputation assignment rule. In particular, considerable ingroup favoritism occurs under the so-called JG assignment rule, whereby observers assign bad reputations to players helping bad players. Ingroup favoritism has been considered to be an evolutionary outcome [25-27,29,33]. The present work supports this general idea. To measure the dependency of ingroup bias on the assignment rule in behavioral experiments may be an interesting challenge.

## Appendices

### A Numerical methods in the case of the homogeneous assignment rule

We prepare a population of *N *= 10^3^DISC players divided into *M* groups of equal size. We consider an *N *×* M* reputation matrix, denoted by ***R ***= (*r*_*i*,*ℓ*_), where *r*_*i*,*ℓ*_∈{G,*B*,*U*} represents the reputation of player *i* (1 ≤* i *≤* N*) in the eyes of the observer in group *ℓ*(1 ≤* ℓ *≤* M*). U represents the unknown reputation. We assume that all the entries of ***R*** are equal to U in the beginning of a run. In a one-shot donation game, we randomly select a player *i* as donor. Then, with probability *θ*/(*N*/*M*−1), we select a recipient *j*(≠*i*) that is in the donor’s group. With probability (1 −* θ*)/(*N *−* N*/*M*), we select a recipient *j* that is in a group different from the donor’s group. When determining the action, the donor refers to *r*_*j*,*k*_, where *k* is the donor’s group. We assume that the donor cooperates when *r*_*j*,*k *_= U. After the game, the observer in each group *ℓ*(1 ≤* ℓ *≤* M*) assigns a new reputation to donor *i* such that *r*_*i*,*ℓ *_= G with probability Φ_G_(*a*,*r*_*j*,*ℓ*_) and *r*_*i*,*ℓ *_= B with probability 1−Φ_G_(*a*,*r*_*j*,*ℓ*_), where *a*∈{C,*D*} is the donor’s action and Φ_G_(*a*,*r*_*j*,*ℓ*_) under each assignment rule is defined in Table 2. When *r*_*j*,*ℓ *_= U, we assume that the observer uses IM; Φ_G_(U,C) = 1−*μ*and Φ_G_(U,D) =* μ*. We set *μ *= 0.01.

After repeating *T *= 10^5^rounds of the donation game, we calculate the fraction of G players in group *k* in the eyes of the observer in group *ℓ*, which is given by $\Phi_{k,\ell }^{*}(G)=\overset{N}{\underset{i=1;\text{player}\;i\;\text{in}\;\mathrm{group}\;k}{\sum }}\delta (r_{i,\ell })/(N/M)$, where *δ*(G) = 1 and *δ*(B) =* δ*(U) = 0. The fractions of G players in the eyes of ingroup and outgroup observers are given by $\Phi_{\mathrm{in}}^{*}(G)=\overset{M}{\underset{k=1}{\sum }}\Phi_{k,k}^{*}(G)/M$ and $\Phi_{\text{out}}^{*}(G)=\overset{M}{\underset{k=1}{\sum }}\overset{M}{\underset{\ell =1,\ell \neq k}{\sum }}\Phi_{k,\ell }^{*}(G)/[M(M-1)]$, respectively. By substituting these quantities in Eqs. (9) and (10), we obtain *ψ* and *ρ*. We average *ψ *and *ρ* over 10^2^ runs of the simulation.

### B Numerical methods in the case of the heterogeneous assignment rule

To analyze heterogeneous populations, we assume that observers in groups 1,2,⋯,*m* adopt JG and those in groups *m* + 1,*m* + 2,⋯,*M*, adopt ST. By applying the procedure explained in Appendix A, we obtain the fraction of G players in group *k* in the eyes of the observer in group *ℓ*, i.e., $\Phi_{k,\ell }^{*}(G)$. The probability that a donor in group *k* helps a recipient is given by

(19) $$\psi_{k}=\theta \Phi_{k,k}^{*}(G)+(1-\theta )\frac{1}{M-1}\overset{M}{\underset{\ell =1,\ell \neq k}{\sum }}\Phi_{\ell ,k}^{*}(G).$$

The probability that a recipient in group *k* is helped by a donor is given by

(20) $$\phi_{k}=\theta \Phi_{k,k}^{*}(G)+(1-\theta )\frac{1}{M-1}\overset{M}{\underset{\ell =1,\ell \neq k}{\sum }}\Phi_{k,\ell }^{*}(G).$$

The ingroup bias of the players in group *k* is given by

(21) $$\rho_{k}=\Phi_{k,k}^{*}(G)-\frac{1}{M-1}\overset{M}{\underset{\ell =1,\ell \neq k}{\sum }}\Phi_{\ell ,k}^{*}(G).$$

The payoff to the players in group *k* is given by

(22) $$\Pi_{k}=-c\psi_{k}+b\phi_{k}.$$

The cooperativeness, ingroup bias, and payoff to the players in groups employing JG and ST are defined by $Q_{\mathrm{JG}}=\overset{m}{\underset{k=1}{\sum }}Q_{k}/m$ and $Q_{\mathrm{ST}}=\overset{M}{\underset{k=m+1}{\sum }}Q_{k}/(M-m)$, respectively, where *Q* represents either *ψ*, *ρ*, or *Π*. We average these quantities over 10^2^runs for each parameter set to generate Figure 7.

## Competing interests

The authors have no competing interests to declare.

## Author’s contributions

MN and NM designed the model. MN derived the analytical and numerical results. MN and NM wrote the paper. Both authors read and approved the final manuscript.
